# The macrophage migration inhibitory factor (MIF)-homologue D-dopachrome tautomerase is a therapeutic target in a murine melanoma model

**DOI:** 10.18632/oncotarget.1560

**Published:** 2013-12-12

**Authors:** Sebastian Kobold, Melanie Merk, Luisa Hofer, Philip Peters, Richard Bucala, Stefan Endres

**Affiliations:** ^1^ Center of Integrated Protein Science Munich (CIPS-M) and Division of Clinical Pharmacology, Department of Internal Medicine IV, Ludwig-Maximilians-Universität München, Munich, Germany, Member of the German Center for Lung Research; ^2^ Internal Medicine, Yale University School of Medicine, New Haven, United States of America

**Keywords:** Cytokine, MIF, cancer, apoptosis

## Abstract

The macrophage migration inhibitory factor (MIF)-homologue D- dopachrome tautomerase (D-DT) recently has been described to have similar functions as MIF. However, the role of D-DT, as opposed to MIF, in tumor biology remains unknown. We hypothesized that D-DT could represent a target for therapeutic interventions in cancer. We analyzed the production of D-DT in the murine melanoma model B16F10 and the murine breast cancer model 4T1 by western blot and ELISA. D-DT was released by tumor cells both in vitro and in vivo. RT-PCR revealed the expression of the D-DT receptor CD74 on both tumor cell lines. Tumor bearing mice had higher serum levels of D-DT compared to healthy controls. Remarkably, knock-down of D-DT by siRNA reduced proliferation of B16F10 cells in BrDU-assay and rendered them more prone to apoptosis induction, as shown by flow cytometry. In vivo neutralization of D-DT by antibodies reduced tumor progression in the B16F10 subcutaneous syngeneic tumor model. In summary, we could show that D-DT and its receptor are expressed in the murine tumors B16F10 and 4T1. Knock-down of D-DT through siRNA or blocking by antibodies reduced proliferation of B16F10 tumor cells. This qualifies D-DT for further evaluation as a therapeutic target.

## INTRODUCTION

The macrophage migration inhibitory factor (MIF) was one of the first cytokines to be described and has since been implicated in many diseases including infectious diseases and cancer [1]. D-Dopachrome tautomerase (DDT) shares 27 % sequence identity with MIF and X-ray analysis have revealed a highly conserved tertiary structure to MIF. However, the biological functions of D-DT have remained unclear for a long time [2, 3]. Recently, we and others have described functional overlaps between MIF and D-DT [4-6]. Both MIF and D-DT bind to the receptor CD74 and induce ERK1/2 phosphorylation, leading to macrophage migration arrest and counterregulation of glucocorticoid-induced immunosuppression [4]. In lung cancer cells, both MIF and D-DT contribute to CXCL8 and VEGF production, two important factors for tumor progression and angiogenesis [5]. Together with the finding that D-DT, as MIF, induces COX-2 expression through stabilization of β-catenin [6], these findings are strongly suggestive of a pro-tumorigenic role of D-DT, as previously demonstrated for MIF [7].

Analysis of clinical samples further showed that D-DT, like MIF, is elevated in sera of patients suffering from ovarian cancer and a correlation between D-DT levels and disease progression has been reported [4].

In cancer cells, MIF seem to play both an autocrine and paracrine role for tumor cell survival and invasiveness [8, 9]. In a MIF-null environment, tumor growth is significantly delayed and part of this effect is mediated by inefficient recruitment of pro-tumoral regulatory cell populations [7, 10]. The important role of MIF in tumor progression has led to the development of MIF-antagonizing or MIF-neutralizing strategies for the treatment of cancer. As a consequence, MIF-inhibitory small molecules and MIF-neutralizing antibodies are currently in preclinical development [10, 11].

Because MIF and D-DT have similar biological functions, we hypothesized that D-DT neutralizing therapeutic strategies may have a similar impact on tumor biology as demonstrated for MIF. We analyzed two aggressive murine tumor models for D-DT secretion in vitro and in vivo. We provide first evidence that mice are a suitable model for the analysis of D-DT in cancer. Furthermore, we demonstrate that the depletion of D-DT via siRNA or neutralization via antibodies results in reduced tumor growth. Therefore these strategies may be developed as a treatment modality against cancer.

## RESULTS

### D-DT is expressed and secreted by two murine cancer cell lines

**Figure 1:**
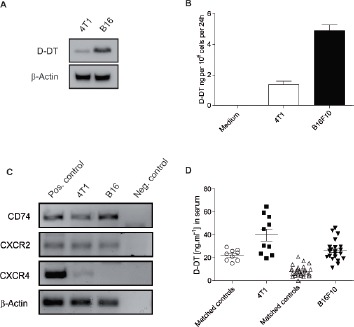
Expression and secretion of D-DT in two murine cancer cell lines (A): Western blot analysis of D-DT levels in B16F10 and 4T1 tumor cells. 50 µg of protein lysate were loaded per lane, β-actin served as a loading control. Results are representative of three independent experiments. (B): D-DT secretion by B16F10 and 4T1 cells. 10^6^ tumor cells were seeded. Supernatants were removed after 24 h and D-DT levels analyzed by ELISA. Results are representative of three independent experiments. Arrows represent the standard error of the mean. (C): Quantitative PCR or known MIF receptors. CD74, CXCR2 and CXCR4 expression was analyzed. β-Actin served as loading control, water and splenocytes served as negative and positive control, respectively. Results are representative of three independent experiments. (D): Serum levels of D-DT in B16F10 and 4T1 tumor-bearing mice. B16F10 tumor bearing mice (n = 22) and matched C57Bl/6 control mice (n = 26) were bled fourteen days after tumor induction. 4T1 tumor bearing mice (n = 10) and matched Balb/c control mice (n = 9) were bled twenty one days after tumor induction. Serum was analyzed by ELISA

To address if D-DT could be used as a target in cancer models, we first analyzed if D-DT was produced by two murine cancer cell lines (the melanoma cell line B16F10 and the breast cancer cell line 4T1). D-DT expression in these cell lines was revealed by Western blot (Figure 1A). Both cell lines secrete significant amounts of D-DT in the cell supernatant as demonstrated by ELISA (Figure 1B). RT-PCR was performed to analyze the known receptors of MIF and D-DT for expression in B16F10 and 4T1 cells. CD74 (known receptor for both) and CXCR2 (known receptor for MIF) but not CXCR4 are expressed on both cell lines (Figure 1C). To further confirm the significance of our findings, we next investigated for the presence of D-DT in tumor bearing mice. Serum was taken from mice with established tumors and D-DT concentration was measured by ELISA. B16F10 tumor bearing mice (n = 22) and 4T1 tumor bearing mice (n = 10) had higher serum levels of D-DT in comparison to wild-type litter mates (n = 26 and 9, respectively). These data indicate a possible autocrine and paracrine role of D-DT in the investigated cancer models.

### D-DT silencing reduces proliferative capacity of murine melanoma cells

**Figure 2:**
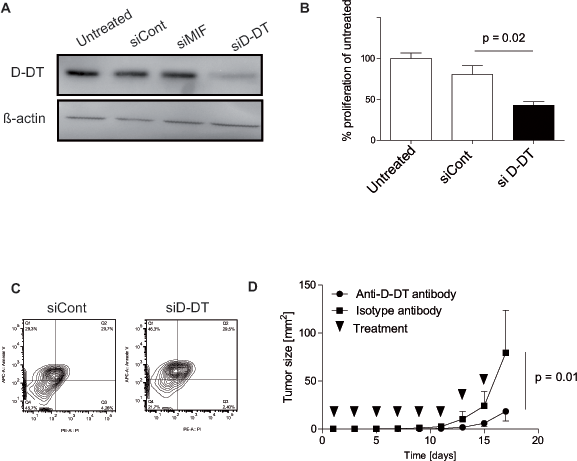
Effects of D-DT knock-down or neutralization on B16F10 tumor cells (A): Analysis of protein depletion after siRNA transfection by Western blot. Transfection was performed twice 48 h apart. Untransfected, control siRNA-transfected and D-DT siRNA-transfected B16F10 tumor cells were analyzed by Western blot 24 h after the last transfection. Results are representative of three independent experiments. (B): Analysis of proliferation by BrdU assay. siRNA for D-DT or control were transfected as in (A) and cells were incubated 48 h after the second transfection for 4 h with BrdU. Proliferation of untreated, control siRNA and D-DT siRNA treated cells were compared. Results are representative of three independent experiments. Arrows represent the standard error of the mean. (C): Apoptosis induction after siRNA transfection through 2 µM staurosporin. Cells were transfected two times, 48 h apart. 24 h after the second transfection, cells were seeded, rested for another 24 h and treated with Staurosporin. Annexin V and PI staining is performed to compare control siRNA and D-DT siRNA-treated B16F10 tumor cells 24 h after staurosporin treatment. Results are representative of three independent experiments. (D): Growth of B16F10 tumors treated with anti-D-DT antibody or control antibody. 250 µg of antibody was administered i.p. every second day (n = 4 per group). Results are representative of two independent experiments. Arrows represent standard error of the mean.

MIF mediates cell proliferation and protects tumor cells from apoptosis [9, 11]. We thus hypothesized a similar role for D-DT, which would qualify D-DT as a therapeutic target. We designed specific siRNA against D-DT which mediated a significant knock-down of D-DT on the protein level in B16F10 cells (Figure 2A). Importantly, no impact on MIF expression could be observed (data not shown). Knock-down of D-DT resulted in a significant decrease in cell proliferation compared to a decoy siRNA (Figure 2B). This decrease in cell proliferation was not due to a reduced cell viability or an increase in cell death (data not shown). Next, we induced apoptosis in B16F10 cells by treating with staurosporin after D-DT silencing. After depletion of D-DT, we observed an increase of apoptosis in comparison to control groups (Figure 2C).

### D-DT neutralization slows tumor progression in vivo

Finally, we tested if D-DT neutralization impacts on tumor growth in vivo. We purified polyclonal D-DT antibodies from immunized rabbit serum by affinity chromatography using the recombinant antigen. Affinity-purified D-DT antibodies or polyclonal rabbit IgG were administered at a dose of 250 µg per mice every second day from tumor injection (n = 4 mice per group). While anti-D-DT antibody injections only resulted in a trend towards longer survival (median survival 23 days for the anti-D-DT-treated group, 18.5 days for the IgG-treated group, results pooled from two experiments; p not significant), treatment significantly slowed tumor progression (Figure 2D).

## DISCUSSION

This report is the first to show expression and secretion of D-DT in the B16F10 melanoma and 4T1 breast cancer model. We also show for the first time that D-DT has a proliferative and anti-apoptotic effect on B16F10 melanoma cells and that targeting of D-DT through siRNA or polyclonal D-DT antibodies may slow tumor progression in this model.

MIF expression has been demonstrated in a wide variety of tumors [1]. In contrast, D-DT expression so far has been evaluated in healthy tissues and only a few cancer models such as lung and colon cancer. In these cell lines a similar expression pattern as MIF has been observed [2, 4]. Therefore, it was not surprising that D-DT is expressed and secreted in the melanoma cell line B16F10 and in the breast cancer cell line 4T1. However, we observed differences between in vitro and in vivo D-DT production: in vitro, D-DT secretion is higher in B16F10 cells, as opposed to in vivo where D-DT serum levels were higher in mice bearing 4T1 tumors. One possible explanation for this discrepancy may be an additional cellular source of D-DT induced by the growing tumors. In fact, immune cells such as myeloid cells are an established source of MIF and D-DT production. These cells play an important role in the biology of these tumor models [12]. The 4T1 tumor model induces a heavy expansion of the myeloid compartment leading to marked splenomegaly and to strong myeloid infiltrates at the tumor sites, both of which may account for the increased D-DT serum levels [12].

Due to the overlapping biological functions of MIF and D-DT, we hypothesized that D-DT may have proliferative and anti-apoptotic effects. The in vitro and in vivo findings confirmed this hypothesis. The findings have important implications, since they constitute the first step in evaluating D-DT as a potential therapeutic target for cancer therapy. Due to the wide spectrum of expression of D-DT in many cancer types analyzed so far, targeting of D-DT may form an attractive option.

In summary, we demonstrate that D-DT may be a suitable target for cancer therapy [11]. Possible formats would be systemic administration of siRNA or monoclonal neutralizing D-DT antibodies, both of which need to be established. It will be interesting to know if our observations in a murine system will also translate in human cancer models and thus warrant further preclinical analysis of D-DT as a therapeutic target.

## MATERIALS AND METHODS

### Cell lines

The murine melanoma cell line B16F10 was cultured in DMEM with 10% fetal bovine serum (FBS, Life Technologies, USA), 1% penicillin and streptomycin (PS) and 1% L-glutamine (all from PAA, Germany). The murine breast cancer cell line 4T1 was provided by M. Wartenberg (Jena, Germany) and cultured in RPMI 1640 with 10% FBS, 1% PS and 1% L-glutamine.

### Animal experiments

Wild type C57BL/6RJ and Balb/cJ mice were purchased from Janvier (St. Berthevin, France). B16F10 and 4T1 tumors were induced by injecting 5 x 105 or 106 tumor cells per mice, respectively. 250 µg of affinity-purified polyclonal rabbit D-DT antibody or polyclonal rabbit IgG were injected i.p. every second day. All animal studies were approved by the local regulatory agency (Regierung von Oberbayern).

### D-DT ELISA

D-DT ELISA was constructed with in-house antibodies and was performed as described [4]. In brief, recombinant D-DT was sent to the SEQLAB-Sequence laboratories (Göttingen, Germany) where it was used to immunize rabbits. Anti D-DT rabbit serum antibodies were recovered using affinity chromatography with binding to recombinant D-DT bound on HiTrap-NHS columns (GE Healthcare, Freiburg, Germany), were eluted and thereafter dialyzed against PBS.

### Western blot

Polyclonal rabbit D-DT antibody was generated and used for Western blot as described [4, 13].

### Reverse transcriptase polymerase chain reaction

Reverse transcription and polymerase chain reaction was performed as described [13] using the following primer sequences: CD74-fwd: 5'-GCA GTG GCT CTT GTT TGA GA-3', CD74-rev: 5'-TTC CTG GCA CTT GGT CAG TA-3', CXCR2-fwd: 2 5'-CAG GAC CAG GAA TGG GAG TA-3', CXCR2-rev: 5'-TCC CCT CCA AAT ATC CCC TA-3', CXCR4-fwd: 5'-TGG AAC CGA TCA GTG TGA GT-3', CXCR4-rev: 5'-GGG CAG GAA GAT CCT ATT GA-3', β-Actin-fwd: 5'-CTA AGG CCA ACC GTG AAA AG-3', β-Actin-rev: 5'-ACC AGA GGC ATA CAG GGA CA-3'

### siRNA lipofection and proliferation assay

siRNA lipofection was performed as described [14] with the following siRNA pairs: D-DT-sense 5'-CUU GGC AGA UCG GAA AG-3' and D-DT-antisense 5'-UUC UUU CCG AUC UGC CAA G-3'. A decoy siRNA served as control: 5'-GCG CAU UCC AGC UUA CGU A-3'. BrdU-proliferation assay (Roche, Mannheim, Germany) was performed according to the manufacturer's instructions.

### Apoptosis assay by annexin V-propidium iodide staining

Analysis of apoptosis was performed as described [13]. Staurosporin (Sigma, Munich Germany) was used as indicated.

### Statistics and data analysis

For statistics GraphPad Prism software, version 5.0b was used. All t-tests were two-tailed and p-values < 0.05 were considered significant. Differences between experimental conditions were analyzed using the unpaired Student's t-test. For in vivo experiments, differences between groups were analyzed using two-way ANOVA with correction for multiple testing.
